# *In vivo* consequences of varying degrees of *OTOA* alteration elucidated using knock-in mouse models and pseudogene contamination-free long-read sequencing

**DOI:** 10.1016/j.gendis.2025.101533

**Published:** 2025-01-18

**Authors:** Ju Ang Kim, Bong Jik Kim, Chung Lee, Go Hun Seo, Hane Lee, Jin Hee Han, Ava Niazi, Joosang Park, Byung Yoon Choi, Sungjin Park

**Affiliations:** a Department of Neurobiology, University of Utah, Salt Lake City, UT 84112, USA; b Department of Otorhinolaryngology, Chungnam National University College of Medicine, Chungnam National University Sejong Hospital, Sejong 30099, South Korea; c Brain Research Institute, Chungnam National University College of Medicine, Daejeon 35015, South Korea; d Department of Pathology, Severance Hospital, Yonsei University College of Medicine, Seoul 03722, South Korea; e 3billion, Inc., Seoul 06193, South Korea; f Department of Otorhinolaryngology–Head and Neck Surgery, Seoul National University Bundang Hospital, Seoul National University College of Medicine, Seongnam 13620, South Korea

Otoancorin (OTOA) is a glycosylphosphatidylinositol (GPI)-anchored protein mediating the attachment of the tectorial membrane (TM) to the spiral limbus (SL) in the inner ear. Homozygous or compound heterozygous mutations in *OTOA* cause autosomal recessive deafness (DFNB22). We performed short-read exome sequencing (SRS) in a 10-month-old boy with sensorineural hearing loss, identifying a potential p.Glu787^∗^ variant in *OTOA*. Interestingly, this variant is common among normal-hearing individuals, leading us to question its pathogenic potential. We generated a knock-in mouse model for this variant and another lacking the C-terminal GPI-anchorage to study the *in vivo* consequences of deleting the C-terminus of Otoa. *Otoa*^*E787*^*^∗^*^*/E787*^*^∗^* mice exhibited reduced transcript expression, TM detachment, and sensorineural hearing loss. Removal of the GPI-anchorage resulted in the loss of surface expression of Otoa and TM detachment, highlighting the importance of the C-terminus. To explain the discrepancy between the pathogenicity of p.Glu787^∗^ in the mouse model and its high allelic frequency in normal-hearing humans, we performed long-read sequencing (LRS) and identified that the variant was in a pseudogene (*OTOAP1*). Whole-genome sequencing revealed an inversion encompassing the 3′ end of *OTOA* in the patient. In summary, we demonstrated the limitations of SRS and confirmed the essential role of the Otoa C-terminus.

A 10-month-old boy (SB391-755) with bilateral moderate sensorineural hearing loss and an autosomal recessive or sporadic inheritance pedigree (Fig. 1A, B) underwent genetic diagnostics, including SRS to narrow down the candidate variants (Fig. S1).^1^^,^^2^ This identified a potential causative variant, c.1765del:p.Gln589Argfs∗55 in *OTOA*, which is predicted to cause premature termination of the *OTOA*.^3^ Given the auditory phenotype and inheritance pattern consistent with DFNB22, we hypothesized a compound heterozygous variant in *OTOA*. The variant, c.2359G > T:p.Glu787^∗^, initially considered a likely deafness-causing variant due to its predicted deletion of the C-terminal GPI anchor region,^3^ has a high minor allele frequency in the Korean population, suggesting it may not be causative (Fig. 1C). This variant's minor allele frequency varied across population databases (Table S1), with significant differences between GnomAD and ExAC. ClinVar classified it as having “conflicting classifications of pathogenicity”. This discrepancy between the molecular biology and genetic data of the variant necessitates further investigation.

**Figure 1:**
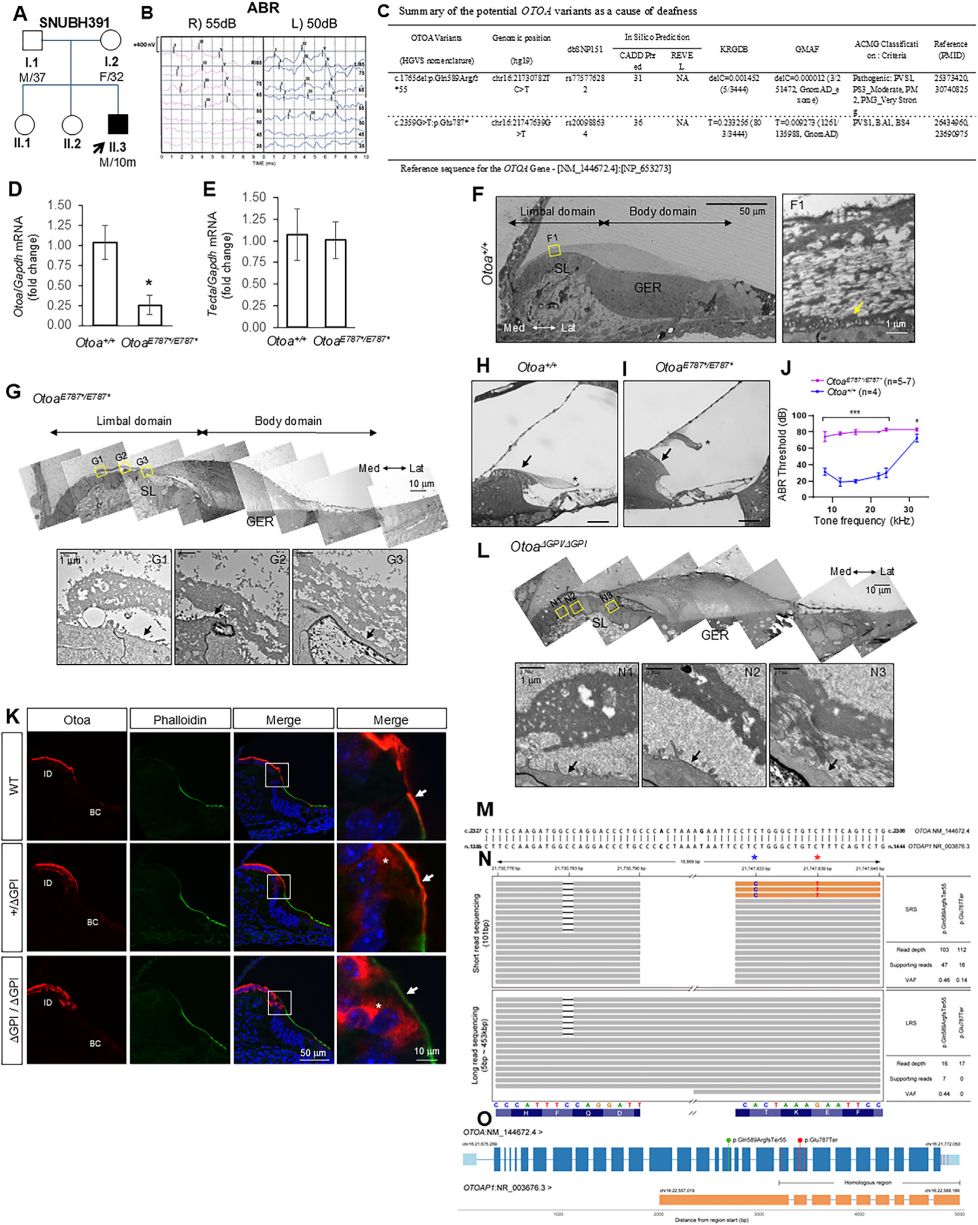
Genetic diagnosis of *OTOA*-related hearing loss in a Korean pedigree and the variant's phenotypes in mice. **(A)** A pedigree showed a sporadic or autosomal recessive inheritance pattern. **(B)** Auditory brainstem response (ABR) demonstrated bilateral moderate hearing loss. The ABR threshold, defined as the lowest level eliciting a wave V response, was 55 dB SPL for the right ear and 50 dB SPL for the left ear. RI, right ear intensity (dB SPL); LI, left ear intensity (dB SPL). **(C)** Conventional short-read sequencing identified potential causative variants in the *OTOA* gene. **(D)** Knock-in mice bearing the p.Glu787^∗^ variant in the *Otoa* gene were generated (*Otoa*^*E787*^*^∗^* allele; Fig. S2A, B). Quantitative reverse transcription PCR of *Otoa* mRNA level relative to *Gapdh* in *Otoa*^*E787*^*^∗^*^*/E787*^*^∗^* cochlea. Error bars: 0.25 ± 0.12. Mean ± standard error of the mean; *n* = 3 animals; ∗*P* = 0.033; two-tailed student's *t*-test. **(E)** Quantitative reverse transcription PCR of *Tecta* mRNA level relative to *Gapdh* in *Otoa*^*E787*^*^∗^*^*/E787*^*^∗^* cochlea. Tecta is required for the attachment of the main body of the tectorial membrane (TM) to the greater epithelial ridge (GER). *Tecta* is expressed normally in *Otoa*^*E787*^*^∗^*^*/E787*^*^∗^* mice, which is consistent with the observed detachment of only the limbal zone of the TM in the mutant mice. Error bars: 1.01 ± 0.21. Mean ± standard error of the mean; *n* = 3 animals; *P* = 0.87; two-tailed student's *t*-test. **(F)** Radial sections of the developing cochlea of wild-type mice at P2. The TM is composed of limbal and body domains along the radial axis. The limbal domain is associated with the apical surface of the spiral limbus (SL), and the body domain is associated with the apical surface of the GER. Dense non-collagenous fibers (a yellow arrow) are associated with the apical surface of the interdental cells. Med, medial; Lat, lateral; F1, transmission electron microscopy of the limbal domain. **(G)** Transmission electron microscopy of the developing TM in *Otoa*^*E787*^*^∗^*^*/E787*^*^∗^* mice at P2. The apical surface of the interdental cells is not associated with dense non-collagenous fibers, and the limbal domain is detached from the SL (black arrows). In contrast, the body domain remains associated with the GER in *Otoa*^*E787*^*^∗^*^*/E787*^*^∗^* mice. **(H)** The radial section of the mature cochlea (P28) was stained with toluidine blue. In the *Otoa*^*+/+*^, TM was attached to the ST (a black arrow), and the marginal band was well organized (an asterisk). Scale bar: 100 μm. **(I)** The radial section of the mature cochlea showed that the TM was detached from the ST at P28 in the *Otoa*^*E787*^*^∗^*^*/E787*^*^∗^* (a black arrow). The marginal band of TM was disorganized in the *Otoa*^*E787*^*^∗^*^*/E787*^*^∗^* (an asterisk). Scale bar: 100 μm. **(J)** The threshold for ABR was significantly elevated in *Otoa*^*E787*^*^∗^*^*/E787*^*^∗^* mice compared with wild-type mice. ∗∗∗*P* < 0.001, ∗*P* < 0.05; unpaired student's *t*-test. **(K)** The localization of the Otoa in wild-type, heterozygous (*Otoa*^*+/ΔGPI*^), and homozygous (*Otoa*^*ΔGPI/ΔGPI*^) mice was monitored by airyscan fluorescence immunohistochemistry. Otoa (red) was expressed in the interdental cells (ID) and border cells (BC). Otoa was expressed mostly on the apical surface of the interdental cells (an arrow), while Otoa was retained within the cytoplasm in homozygous mice (an asterisk). In heterozygous mice, both surface-expressed (an arrow) and cytoplasmic pools (an asterisk) of Otoa proteins were observed. Knock-in mice lacking the GPI-anchorage of Otoa were generated (*Otoa*^*ΔGPI*^ allele; Fig. S2C, D). **(L)** Transmission electron microscopy of the developing TM in *Otoa*^*ΔGPI/ΔGPI*^ mice at P2. The apical surface of the interdental cells is not associated with dense non-collagenous fibers, and the limbal domain was detached from the ST (arrows in L1, L2, and L3). In contrast, the body domain remained associated with the GER in *Otoa*^*ΔGPI/ΔGPI*^ mice. **(M)** The partial result of nucleotide BLAST around p.Glu787^∗^ between *OTOA* (Query ID: NM_144672.4) and *OTOAP1* (subject ID: NR_003676.3). **(N)** Illustrated Integrative Genomics Viewer (IGV) captured images between short read sequencing (top) and long read sequencing (bottom) of *OTOA* p.Gln589Argfs^∗^55 (c.1765delC) (left, a blue asterisk) and p.Glu787^∗^ (c.2359G > T) (right, a red asterisk) with information of sequencing data (right table). The orange reads indicated that *OTOAP1* was sequenced but aligned to *OTOA*. **(O)** Depicting the correspondence of exon (blue box) levels between *OTOA* and *OTOAP1* (orange box), including detected variant (lollipop shape) and 7 mismatched sequences (red vertical line) in the homologous region that could be identified as a false positive variant call at short read sequencing. Notably, only differences occurring in exon 22 are relevant as the variant in exon 21 is a synonymous variant and those in exon 29 are in the 3′UTR.

To determine the pathogenicity of the *OTOA* p.Glu787^∗^ variant, we generated a disease-mimicking variant in mice by CRISPR/Cas-9 (*Otoa*^*E787*^*^∗^* allele; Fig. S2A, B). Notably, in a real-time reverse transcription PCR analysis, the *Otoa* mRNA level in *Otoa*^*E787*^*^∗^*^*/E787*^*^∗^* mice was significantly reduced, while the expression level of *Tecta*, an inner ear-specific TM glycoprotein, remained unchanged (Fig. 1D, E). These observations showed that the premature termination of *Otoa* p.Glu787^∗^ caused instability of its transcript in mice.

Next, we examined the developing TM ultrastructure by transmission electron microscopy at postnatal day 2 (P2). In wild-type mice, the TM was associated with the apical surface of the cochlear supporting cells (Fig. 1F). Dense non-collagenous fibers also connected the limbal zone of the TM to the apical surface of the interdental cells within the SL (Fig. 1F, arrow). In *Otoa*^*E787*^*^∗^*^*/E787*^*^∗^* mice, while the body domain of the TM was normally connected to the apical surface of greater epithelial ridge cells, the limbal domain was detached from the SL (Fig. 1G). The dense non-collagenous fibers were organized within the limbal domain but were not associated with the interdental cells, indicating that Otoa recruits these fibers to the apical surface of interdental cells. Examination of the mature TM by the semi-thin sectioning showed that in wild-type mice, the limbal domain of the TM was attached to the SL (Fig. 1H, arrow). In *Otoa*^*E787*^*^∗^*^*/E787*^*^∗^* mice, the TM was completely detached from the SL (Fig. 1I, arrow), the marginal band was deformed (Fig. 1I, asterisk), and the auditory brainstem response threshold was significantly elevated (Fig. 1J) as in null mice.^4^

While the p.Glu787^∗^ reduces transcript levels in mice, the regulation of *OTOA* mRNA stability may differ in humans. Since the null phenotype of *Otoa*^*E787*^*^∗^*^*/E787*^*^∗^* mice can be primarily attributed to decreased OTOA expression rather than disrupted protein function, we cannot rule out the possibility that the downstream sequence of p.Glu787∗ might be non-essential for protein function. To directly assess the role of the OTOA C-terminus, we specifically deleted the extreme C-terminus, including the GPI-attachment sequence following the GPI-attachment site (ω -site) (*Otoa*^*ΔGPI*^ allele; Fig. S2C, D).

Airyscan high-resolution imaging of Otoa immunooumigahistochemistry showed wild-type Otoa on the apical surface of the interdental cells, marked by phalloidin staining at P2 (Fig. 1K, arrows). We anticipated Otoa lacking GPI-anchorage (Otoa^ΔGPI^) to be secreted from cells as shown in other GPI-anchored proteins.^5^ However, it was retained within the cytoplasm (Fig. 1K, asterisks). The overall intensity of Otoa in the interdental cells of *Otoa*^*ΔGPI/ΔGPI*^ mice was comparable to that in wild-type mice (Fig. 1K). In *Otoa*^*+/ΔGPI*^ mice, both surface-expressed and cytoplasmic pools of Otoa were observed, indicating that GPI-anchorage is essential for the surface expression of Otoa. Transmission electron microscopy analysis of the developing TM in *Otoa*^*ΔGPI/ΔGPI*^ mice at P2 showed null phenotypes similar to *Otoa*^*E787*^*^∗^*^*/E787*^*^∗^* mice, with a significant elevation of auditory brainstem response threshold (Fig. 1L; Fig. S3A, B). As previously reported in the heterozygous Otoa null mice,^4^ the TM of *Otoa*^*+/E787*^*^∗^* and *Otoa*^*+/ΔGPI*^ mice displayed normal morphology (not shown). These observations indicate that the Otoa C-terminus is essential and its truncation is highly pathogenic in mice. Thus, we reasoned that the high prevalence of p.Glu787^∗^ detected by SRS in a normal-hearing population would be unlikely if this variant existed within the true *OTOA*.

Consequently, we revisited the genetic diagnosis of our patient to check for pseudogene (*OTOAP1*) involvement. Aligning *OTOA* and *OTOAP1* revealed that T at the 1417th position of *OTOAP1* non-coding RNA of *OTOAP1* (wild allele) corresponded to G at the 2359th position of *OTOA* mRNA (altered allele) (Fig. 1M), suggesting SRS might have misinterpreted the *OTOA* due to pseudogene contamination. LRS confirmed that p.Glu787^∗^ in *OTOA* was not present in reads from our patient covering both non-homologous and homologous regions. The Integrative Genomic Viewer showed a significantly lower detection frequency of p.Glu787∗ (0.14) compared with that of p.Gln589Argfs∗55 (0.46) among the total reads in SRS, and no detection of the read for p.Glu787∗ in LRS from our patient (Fig. 1N). Conversely, the p.Gln589Argfs∗55 variant was consistently observed in both SRS and LRS, with about 0.5 of the detection rate of the altered allele. Basic local alignment search tool (BLAST) analysis around G at position 2359 of *OTOA* matched well with T at position 1417 of *OTOAP1*, revealing seven mismatched sequences containing p.Glu787^∗^ in the homologous region between *OTOA* and *OTOAP1* (Fig. 1O and Table S2).

Whole-genome sequencing investigated the cause of sensorineural hearing loss in the pedigree, focusing on a potential second variant or structural variation in *OTOA*. No additional small variants were found. However, whole-genome sequencing revealed a large inversion on chromosome 16p12.2, encompassing the 3′ end of the *OTOA* gene. This inversion spanned exons 17 and 18 of *OTOA* and extended across two downstream genes, NPIPB4 and UQCRC2. Its breakpoints were located in the deep intronic regions of both the *OTOA* and *UQCRC2* with breakpoints in deep intronic regions, making it copy-neutral and undetectable by exome sequencing (Fig. S4). The patient was diagnosed with sensorineural hearing loss likely due to a combination of p.Gln589Argfs∗55 and a large inversion in *OTOA*.

Taken together, we identified the cause of hearing loss in a pedigree by addressing the limitations of SRS with LRS. We conducted auditory phenotyping of *OTOA* knock-in mice modeling the p.Glu787∗ variant and lacking a GPI-anchor. These mutations caused *Otoa* transcript instability and trafficking defects, respectively, providing mechanistic insights into DFNB22. The loss of the Otoa C-terminus and GPI-anchorage could be directly linked to DFNB22 pathogenesis. LRS and whole-genome sequencing enabled a complex genetic diagnosis involving pseudogene contamination and large inversions in *OTOA*.

## Ethics declaration

The human research in this study was approved by the Institutional Review Board of Seoul National University Bundang Hospital (IRB-B-1007-105-402), and written informed consent was obtained from all subjects. The animal research in this study was approved by the Institutional Animal Care and Use Committee of the University of Utah (Protocol 21–02004).

## Author contributions

**Ju Ang Kim:** Writing – original draft, Visualization, Investigation, Formal analysis. **Bong Jik Kim:** Writing – review & editing, Writing – original draft, Investigation, Funding acquisition, Formal analysis, Conceptualization. **Chung Lee:** Visualization, Resources. **Go Hun Seo:** Visualization, Resources. **Hane Lee:** Visualization, Resources. **Jin Hee Han:** Formal analysis, Data curation. **Ava Niazi:** Validation, Resources. **Joosang Park:** Validation, Resources. **Byung Yoon Choi:** Writing – review & editing, Supervision, Project administration, Funding acquisition, Conceptualization. **Sungjin Park:** Writing – review & editing, Supervision, Project administration, Methodology, Funding acquisition, Conceptualization.

## Funding

This work was supported by the National Research Foundation of Korea (NRF) grant funded by the Korean government (MSIT) (No. 2021R1C1C1007980 to B.J.K.), Chungnam National University Sejong Hospital Research Fund, 2022, and Chungnam National University (to B.J.K.). This study was supported by the Basic Science Research Program through the NRF, funded by the Ministry of Education (No. 2021R1A2C2092038 to B.Y.C.), Bio Core Facility Center program (No. NRF-2022M3A9G1014007 to B.Y.C.), the Basic Research Laboratory program through the NRF, funded by the 10.13039/501100002701Ministry of Education (No. RS-2023-0021971031482092640001 to B.Y.C.), and the Technology Innovation Program (No. K_G012002572001 to B.Y.C.) funded By the Ministry of Trade, Industry & Energy (MOTIE, Korea). This study is also funded by SNUBH (Seoul National University Bundang Hospital) intramural research fund (No. 13-2022-0010,02-2017-0060, 16-2023-0002, 13-2023-0002,16-2022-0005, 13-2024-0004, and 13-2017-0013 to B.Y.C.). This work was supported by the National Institute on Deafness and Other Communication Disorders (NIDCD), part of the US National Institutes of Health (No. R01DC018814 to S.P.).

## Conflict of interests

The authors declared no conflict of interests.

## Figures and Tables

**Figure S1:**
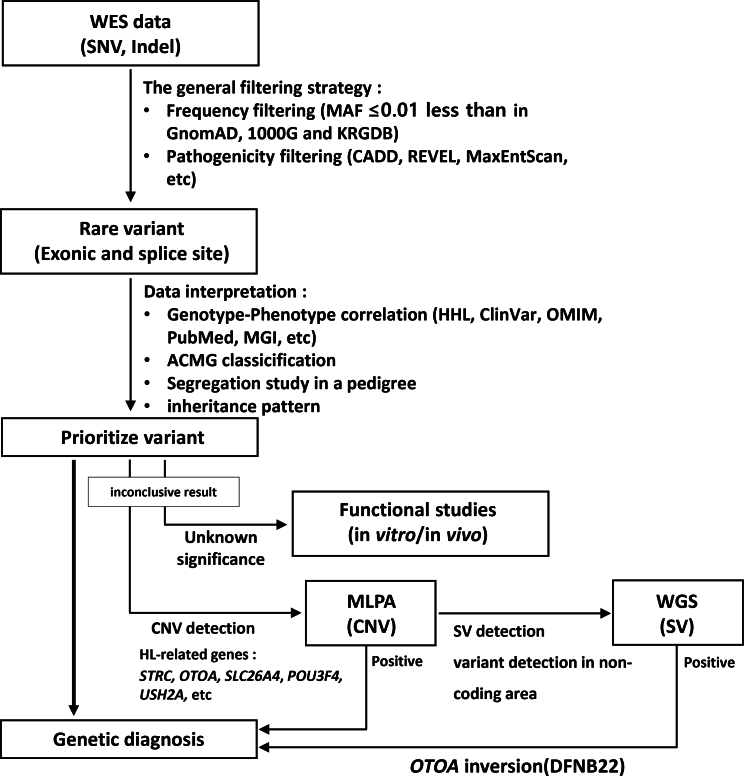
Genetic diagnosis flow. Multiple bioinformatics tools were adopted to narrow down the candidate causative variants, and then clinical information including inheritance pattern/online genotype-phenotype databases, was used to finalize the genetic diagnosis. SNV, single nucleotide variant; Indel, insertion-deletion; MAF, minor allele frequency; SV, structural variant; CADD, Combined Annotation Dependent Depletion (https://cadd.gs.washington.edu/); REVEL, Rare Exome Variant Ensemble Learner (https://sites.google.com/site/revelgenomics/); KRGDB, Korean Reference Genome Database (http://coda.nih.go.kr/coda/KRGDB/index.jsp); GnomAD, The Genome Aggregation Database (https://gnomad.broadinstitute.org/); HHL, Hereditary Hearing Loss Homepage (https://hereditaryhearingloss.org/). ACMG/AMP 2018 guideline is available at http://wintervar.wglab.org/. MaxEntScan is accessible at http://hollywood.mit.edu/burgelab/maxent/Xmaxentscan_scoreseq.html.

**Figure S2:**
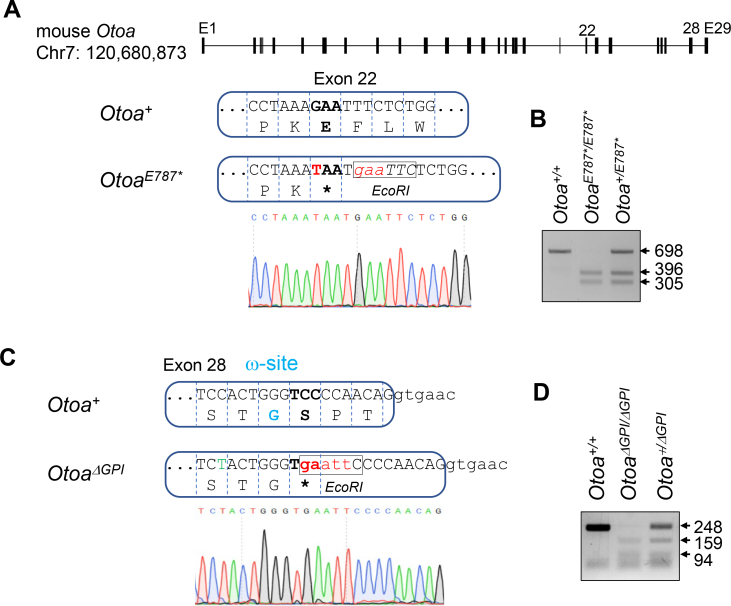
Generation and characterization of *Otoa* variant mice. (A) Schematic of *Otoa* gene and the CRISPR/Cas9 editing site located in exon 22 in the C57Bl/6J mouse. p.Glu787∗ (NP_647471.1) was replaced with a stop codon (∗), followed by an EcoR1 site. Sanger sequencing confirmed the expected sequence replacement with no additional mutations. (B) PCR amplification followed by EcoRI digestion showed a 698 bp band from the wild-type allele and 396 and 305 bp bands from the *Otoa^E787∗^* allele. (C) The p.Ser1114 (NP_647471.1) following the omega-site (ω-site: p.Gly1113) in exon 28 was replaced with a stop codon (∗), followed by an EcoR1 site. A silent mutation was introduced on p.Ser1111 to remove the PAM sequence of gRNA target. Sanger sequencing confirmed the expected sequence replacement with no additional mutations. (D) PCR amplification followed by EcoRI digestion showed a 248 bp band from the wild-type allele and 159 and 94 bp bands from the *Otoa^E787∗^* allele.

**Figure S3:**
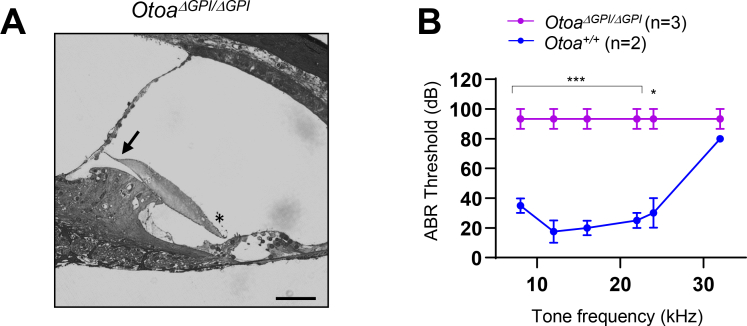
Characterization of *Otoa^ΔGPI/ΔGPI^* mice. (A) Radial sections of the mature cochlea (P28) stained with toluidine blue showed that the TM was detached from the spiral limbus at P28 in the *Otoa^ΔGPI/ΔGPI^* (an arrow). The marginal band was disorganized in the *Otoa^ΔGPI/ΔGPI^* TM (an asterisk). Scale bar: 100 μm. (B) The threshold for auditory brainstem response (ABR) was significantly elevated in OtoaΔGPI/ΔGPI mice. ^∗∗∗^*P* < 0.001, ^∗^*P* < 0.05; unpaired student's t-test.

**Figure S4:**
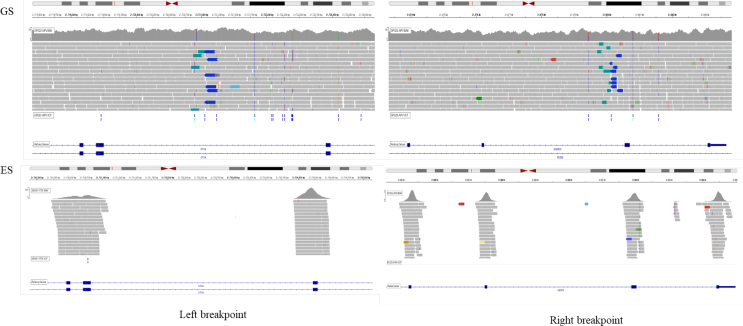
Whole-genome sequencing information. Whole-genome sequencing revealed a large inversion on chromosome 16p12.2, encompassing the 3′ end of the *OTOA* gene [NC_000016.10:g.[21721049_21721051del;21721052_21980114inv;21980115del] (GRCh38)]. The inversion was identified using blue and cyan colored reads in the Integrative Genomics Viewer (IGV) view of the variant. The figure shows the coverage data of genome sequencing (GS) in the top panel and exome sequencing (ES) in the bottom panel. The top panel shows many reads aligning across the breakpoints, while the bottom panel shows no reads in the intron region. The left breakpoint was identified within the intron 17 of the *OTOA* gene and the right breakpoint was identified within the intron 12 of the *UQCRC2* gene from whole-genome sequencing.
