# Supplementary material for: In vivo consequences of varying degrees of OTOA alteration elucidated using knock-in mouse models and pseudogene contamination-free long-read sequencing
Source: Genes Dis. 2025 Jan 18;12(3):101533. doi: 10.1016/j.gendis.2025.101533 (PMC11815939; doi:10.1016/j.gendis.2025.101533)
Supplement: Multimedia component 1 [file mmc1.docx]

**MATERIALS and METHODS**

**Conventional genetic diagnosis of the subject**

A 10-month-old boy (SB391-755) visited our clinic, presenting with bilateral moderate SNHL (55 dBHL) measured by auditory brainstem response (ABR) threshold testing. A pedigree showed an autosomal recessive or sporadic inheritance pattern (**Fig. 1A-B**). Genomic DNA samples from the blood of the proband and his parents were collected and were analyzed for genetic diagnosis. First, 11 recurring variants of five deafness genes were screened ^1^, followed by multiplex ligation-dependent probe amplification (MLPA) of the *STRC* and *OTOA* genes according to a protocol previously proposed by our research group ^2^. These steps did not identify candidate variants or CNVs. Next, we moved to exome sequencing using conventional short-read sequencing (SRS). The detailed filtering steps using bioinformatics tools have been described previously ^3^. Routine SRS resulted in inconclusive results: two potential candidate variants of *OTOA*: p.Gln589Argfs*55 (a known pathogenic variant) and p.Glu787* (a previously reported variant) (**Fig. 1C**) ^4^. The p.Glu787* variant is expected to disrupt GPI-anchorage, which would likely be related to the pathogenesis of hearing loss. However, the high minor allele frequency (0.23) of p.Glu787* in the Korean population strongly suggests it is not pathogenic and this variant’s MAF varies across population databases (**Table S1**). To determine whether p.Glu787* is located in the pseudogene *OTOAP1* rather than in *OTOA*, and thus lacks pathogenic potential, we performed LRS. Additionally, we generated a knock-in (KI) mouse model of p.Glu787* to directly assess its pathogenicity in an *in vivo* model.

**Auditory Brainstem Response (ABR) measurements**

ABR was measured to evaluate the hearing status as was described^5^. In brief, the ABR threshold was defined as the minimum sound pressure level (SPL) that elicited a wave V response. The stimulus levels ranged from 85 to 35 dB SPL in 5 to 10 dB intervals. More specifically, when the wave V was clearly visible, we reduced the stimulus intensity in 10 dB steps. When the wave V became very small or was no longer visible, we decreased the stimulus intensity in 5 dB steps to more accurately determine the threshold. (**Fig. 1B**).

**Long-read exome sequencing and whole-genome sequencing**

We aligned long reads to the human genome reference (GRCh19) using the MEM algorithm of the Burrows-Wheeler Aligner (version 0.7.17), with the read type option (-x) set to "pacbio," which is optimized for aligning long-read sequencing data from PacBio. Small variants were called using HaplotypeCaller from GATK (version 4.1.0.0). Additionally, the absence of p.Glu787* in OTOA was directly confirmed through IGV. Whole genome sequencing (WGS) was conducted at 3billion, Inc., using high molecular weight genomic DNA extracted from a whole blood sample. The library was prepared using the TruSeq DNA PCR-Free kit, and sequencing was performed using NovaSeq 6000 (Illumina, San Diego, CA, USA). Alignment to the GRCh38 human reference genome was performed using BWA-MEM2, and samtools v1.15 was utilized for sorting BAM files and marking duplicates ^6;7^ . Recalibration and variant calling for single nucleotide variants (SNVs) and small insertion/deletion variants (indels) were performed using GATK v4.2 ^8^. Structural variants (SVs), including CNVs, were called using MANTA v1.6.0, and 3bCNV, an internally developed tool by 3billion ^9^. ExpansionHunter v5.0.0 for calling repeat expansion variants and MELT v2.2.2 for calling mobile element insertion were used ^10;11^. Variants were annotated, filtered, and classified using EVIDENCE v3.2, which incorporates the Ensembl Variant Effect Predictor for annotation and follows the American College of Medical Genetics and Genomics guidelines for classification ^12;13^. The filtered and classified variants were manually reviewed by medical geneticists and physicians. The most likely variants that could explain the patient’s phenotype were selected for reporting.

**CRISPR/Cas9 design and mutant mice generation**

*Otoa^E787*^* and *Otoa^ΔGPI^* mouse lines (C57Bl6/J background) were generated by CRISPR/Cas9-based genome editing at the University of Utah Transgenic and Gene Targeting Core. *Tecta* is located in an autosome (chromosome 7).

1. *Otoa^E787*^* mouse line: Two guide RNAs (gRNAs) targeting 5’-acccaccttcaggcattaagcgg-3’ (targeting an upstream intron of E22) and 5’- aggcttgaaggcttgatgagtGg-3’ (targeting the downstream intron of E22) were used. A 519-bp-long, single-strand oligo donor (ssODN) for *Otoa^E787*^* was synthesized, comprising a left-homology arm (75 bp), a right-homology arm (75 bp), a mutation to change each protospacer adjacent motifs (PAM) in the intron, a point mutation to change E787 (GAA) to Stop (TAA), and a unique EcoR1 (GAATTC) site. Then, 2 μL of a mixture containing gRNAs (30 ng/μL), the donor (50 ng/μL), and Cas9 protein (100 ng/μL) was injected per C57BL6/J pronuclear embryo. After incubation overnight, the injected embryos were implanted into the oviducts of pseudopregnant females.

2. *Otoa^ΔGPI^* mouse line: an antisense gRNA targeting 5’- ttcacCTGTTGGGGACCCAGTGG-3’ was used to target the ω-site in exon 28. A 105-bp-long ssODN was synthesized, comprising a left-homology arm (45 bp), a right-homology arm (47bp), a silent mutation to change PAM on p.Ser1111 (TCC to TCT), a point mutation to change p.Ser1114 (TCC) to Stop (TGA) and a unique EcoR1 (GAATTC) site. Then, 2 μL of a mixture containing gRNA (30 ng/μL), the donor (50 ng/μL), and Cas9 protein (100 ng/μL) was injected per C57BL6/J pronuclear embryo. After incubation overnight, injected embryos were implanted into the oviducts of pseudopregnant females.

**Genotyping**

P0 mouse tails (3 mm) were collected in 1.5-mL microcentrifuge tubes and combined with 300 µL of tissue digestion buffer (10 mM Tris, pH 8.0, with 100 mM NaCl, 0.1 mM EDTA, 0.5% SDS, and 15 µL of proteinase K [Fisher Scientific, 25530049, 20 mg/mL]). After overnight incubation at 60°C, the mixture was further treated with 200 µL of 3 M NaCl for 10 min. Subsequently, the mixture was chilled on ice for 10 min, and the supernatant was collected through centrifugation (6,000 rpm for 10 min at room temperature). A genomic DNA pellet was obtained by treatment with 1 mL of 100% ethanol followed by centrifugation at 13,000 rpm at 4°C for 30 min. The pellet was washed with 70% ethanol and dissolved in 100 µL of distilled water. Subsequently, 100 ng of genomic DNA was added to the PCR reagent kit (DreamTaq DNA Polymerase, Invitrogen, EP0703) in a total volume of 15 µL and processed according to the manufacturer's instructions. The PCR conditions were as follows: 94°C for 30 sec, 60°C for 30 sec, and 72°C for 45 sec for 34 cycles. The initial denaturing step and the final extension condition were 94°C for 3 min and 72°C for 10 min, respectively.

For *Otoa^E787*^* genotyping, both *Otoa^WT^* and *Otoa^E787*^* alleles generated a 698-bp band using the *Otoa^E787*^* primer set of *Otoa^E787X^*F: 5’- TTGGCAATCTGATGGGTG-3’ and *Otoa^E787X^*R: 5’-CTGTGATGCTTGTTCCTG-3’ (at 60°C, targeting exon 22, an edited sequence of *p.Glu787** in NCBI number: NP_647471.1). The overnight EcoRI digestion generates 396 bp and 305 bp fragments for *Otoa^E787*^*. For *Otoa^ΔGPI^* genotyping, both *Otoa^WT^* and *Otoa^ΔGPI^* alleles generated a 248-bp band using the *Otoa^ΔGPI^* primer set of *Otoa^ΔGPI^*F: 5’-CTCAGAATGCAGCCTCTG-3’ and *Otoa^ΔGPI^*R: 5’-GTCAACATCACCTGGCCCT-3’ (at 60°C, targeting exon 28, an edited sequence of *p.Gly1114X* in the NCBI number: NP_647471.1). Overnight EcoRI digestion generated 159-bp and 94-bp fragments for *Otoa^ΔGPI^*.

**Immunohistochemistry**

Mice were euthanized under anesthesia using isoflurane, and the cochlea was dissected from the temporal bone. A small hole was created at the apex, and the oval and round windows were opened. The samples were fixed in 4% PFA in PBS overnight at 4°C. Afterward, the cochlea was incubated in a 50% OCT (Ted Pella, 27050) and 30% sucrose solution in PBS (v/v) for 1 hour at room temperature, following several PBS washes. It was then transferred to an embedding mold (Polysciences, 18646A-1) filled with 100% OCT and subsequently frozen on dry ice. The frozen cochlea sections were cut to a thickness of 7 µm and mounted on glass slides (Fisher Scientific, 22-037-246). Tissue sections were blocked in a solution containing 10% normal goat serum (NGS) in PBS-T (0.25% Triton X-100 in PBS) for 1 hour at room temperature. This was followed by an overnight incubation with an α-Otoa antibody produced in rabbits (1:200) (Biorbyt, orb2853) in a dilution solution (5% NGS in PBS-T) at 4°C. After three washes with PBS-T, the sections were incubated with Cy™3 AffiniPure Donkey α-Rabbit IgG (H+L) (1:500) (Jackson Immuno Research, 711-165-152), Alexa Fluor™ 488 Phalloidin (1:1000) (ThermoFisher, A12379), and Hoechst 33342 (1:20,000) (Invitrogen, H3570) at room temperature for 1 hour. Finally, the tissues were mounted with Fluromount-G® (SouthernBiotech, 0100-01) after three PBS-T washes and cover-slipped for imaging.

**Semi-thin sections**

The cochlea was dissected as described above. Then, the samples were processed for imaging as previously described ^14^.

**Tissue processing for scanning and transmission electron microscopy**

The preparation of cochlear tissue for both scanning electron microscopy (SEM) and transmission electron microscopy (TEM) followed the method demonstrated in semi-thin sections. For SEM, the cochlear coil, once fixed, was carefully detached from the bony shell, washed, and dehydrated in a series of steps before undergoing critical point drying using a Pelco CPD2. The samples were then affixed to a stub with a carbon adhesive tab (Ted Pella, 16084-2) and coated with a gold/palladium layer using a sputter coater to prepare them for imaging. For TEM, the plastic-embedded tissues were cut radially into sections 100 nm thick using a diamond knife on an Ultramicrotome. These sections were placed on 200 mesh copper grids and sequentially stained with saturated aqueous uranyl acetate and Reynolds’ Lead Citrate for imaging.

**Quantitative PCR (qPCR)**

At P0, the cochlea was dissected to extract RNA using TRI Reagent™ Solution (Invitrogen™, AM9738). cDNA was synthesized using 1 µg of total RNA in a reverse transcription (RT) reaction using the PrimeScript™ High Fidelity RT-PCR Kit (Takara, R022A) in a 20-µL volume, following the manufacturer's instructions. Specifically, 0.5 µL of the cDNA was mixed in a 23 µL of SYBR™ Select Master Mix (Applied Biosystems™, 4472908) with each set of primers: *Otoa* primers 5’-ACCTGTGAGTGGCGCTACGATG-3’ (JA31) and 5’-GTGACTTCATCCAGGACCTTAGG-3’ (JA32) (60°C, 143 bp, NCBI number: AY055122), or *Tecta* primers 5’-GCTTCCTCTTCCACTTCCAAG-3’ (DK88) and 5’-GCCCAAAATCTGTTTCCACG-3’ (DK89) (58°C, 300 bp, NCBI number: NM_009347.4). Additionally, the primers for *Gapdh* were 5’-CTGGAGAAACCTGCCAAGTA-3’ (DK50) and 5’- AGTGGGAGTTGCTGTTGAAG -3’ (DK51) (58°C, 130 bp, NCBI number: NM_001289726.2). Each set of these primers, along with the cDNA, was mixed with 23 µL of SYBR™ Select Master Mix (Applied Biosystems™, 4472908) for a total volume of up to 25 µL. Gene expression analysis was carried out using PCR with 40 cycles (20 sec at 95°C, 20 sec at the annealing temperature specific to each primer set, and 40 sec at 72°C) on a Life Technologies QuantStudio 12K Flex instrument. The mRNA levels of *Otoa* and *Tecta* were normalized to the levels of *Gapdh*.

**Imaging**

Images for immunohistochemistry were captured using a Zeiss 880 Airyscan confocal microscope equipped with Zen Black software at the University of Utah Cell Imaging Core. SEM and TEM images were obtained using the FEI Quanta 600 FEG and the FEI Tecnai 12 transmission electron microscope, respectively, at the University of Utah Electron Microscopy Core. Images for semi-thin sections were captured with a Leica DM2500 optical microscope using Leica Las software V3.8. All images were processed and analyzed using Image J (version 1.54f).

**References**

1. Han KH, Kim AR, Kim MY, et al. Establishment of a Flexible Real-Time Polymerase Chain Reaction-Based Platform for Detecting Prevalent Deafness Mutations Associated with Variable Degree of Sensorineural Hearing Loss in Koreans. *PLoS One.* 2016;11(9):e0161756.

2. Kim BJ, Oh DY, Han JH, et al. Significant Mendelian genetic contribution to pediatric mild-to-moderate hearing loss and its comprehensive diagnostic approach. *Genet Med.* 2020;22(6):1119-1128.

3. Kim BJ, Kim AR, Lee C, et al. Discovery of CDH23 as a Significant Contributor to Progressive Postlingual Sensorineural Hearing Loss in Koreans. *PLoS One.* 2016;11(10):e0165680.

4. Laurent S, Gehrig C, Nouspikel T, et al. Molecular characterization of pathogenic OTOA gene conversions in hearing loss patients. *Hum Mutat.* 2021;42(4):373-377.

5. Jang MW, Oh DY, Yi E, et al. A nonsense TMEM43 variant leads to disruption of connexin-linked function and autosomal dominant auditory neuropathy spectrum disorder. *Proc Natl Acad Sci U S A.* 2021;118(22).

6. Li H, Handsaker B, Wysoker A, et al. The Sequence Alignment/Map format and SAMtools. *Bioinformatics.* 2009;25(16):2078-2079.

7. Vasimuddin M, Misra, S., Li, H., & Aluru, S. Efficient Architecture-Aware Acceleration of BWA-MEM for Multicore Systems. *2019 IEEE International Parallel and Distributed Processing Symposium (IPDPS).* 2019:314-324.

8. McKenna A, Hanna M, Banks E, et al. The Genome Analysis Toolkit: a MapReduce framework for analyzing next-generation DNA sequencing data. *Genome Res.* 2010;20(9):1297-1303.

9. Chen X, Schulz-Trieglaff O, Shaw R, et al. Manta: rapid detection of structural variants and indels for germline and cancer sequencing applications. *Bioinformatics.* 2016;32(8):1220-1222.

10. Dolzhenko E, Deshpande V, Schlesinger F, et al. ExpansionHunter: a sequence-graph-based tool to analyze variation in short tandem repeat regions. *Bioinformatics.* 2019;35(22):4754-4756.

11. Gardner EJ, Lam VK, Harris DN, et al. The Mobile Element Locator Tool (MELT): population-scale mobile element discovery and biology. *Genome Res.* 2017;27(11):1916-1929.

12. Richards S, Aziz N, Bale S, et al. Standards and guidelines for the interpretation of sequence variants: a joint consensus recommendation of the American College of Medical Genetics and Genomics and the Association for Molecular Pathology. *Genet Med.* 2015;17(5):405-424.

13. Seo GH, Kim T, Choi IH, et al. Diagnostic yield and clinical utility of whole exome sequencing using an automated variant prioritization system, EVIDENCE. *Clin Genet.* 2020;98(6):562-570.

14. Kim DK, Kim JA, Park J, Niazi A, Almishaal A, Park S. The release of surface-anchored alpha-tectorin, an apical extracellular matrix protein, mediates tectorial membrane organization. *Sci Adv.* 2019;5(11):eaay6300.
