# Supplementary material for: In vivo consequences of varying degrees of OTOA alteration elucidated using knock-in mouse models and pseudogene contamination-free long-read sequencing
Source: Genes Dis. 2025 Jan 18;12(3):101533. doi: 10.1016/j.gendis.2025.101533 (PMC11815939; doi:10.1016/j.gendis.2025.101533)
Supplement: Multimedia component 2 [file mmc2.docx]

**Supplementary Table 1**. c.2359G>T: p.Glu787* variant of *OTOA* in population databases

| **Genomic Position: Change (GRCh37/hg19)** | **HGVS** | | **Insilico Predictions** | | **Minor Allele Frequency** | **dbSNP ID** | **ClinVar** |
| --- | --- | --- | --- | --- | --- | --- | --- |
|  | **Nucleotide**  **change** | **Amino Acid**  **change** | **CADD Phred** | **GERP++** |  |  |  |
| **Chr16:21747639 G>T** | **c.2359G>T** | **p.Glu787Ter** | **39.00** | **4.18** | **T=0.009273 (1261/135988, GnomAD)**  **T=0.001220 (147/120532, ExAC)**  **T=0.29355 (8295/28258, 14KJPN)**  **T=0.00886 (225/25394, ALFA)**  **T=0.29081 (4874/16760, 8.3KJPN)**  **T=0.0617 (395/6404, 1000G_30x)**  **T=0.2690 (786/2922, KOREAN)** | **rs200988634** | **Conflicting classifications of pathogenicity**  **Pathogenic; Likely pathogenic; Uncertain significance** |

The Refseq transcript used for OTOA accession number NM_144672.4; Refseq protein accession number NP_653273.

HGVS: Human Genome Variation Society (https://www.hgvs.org/)

CADD: Combined Annotation Dependent Depletion (https://cadd.gs.washington.edu/)

GERP++: Genomic Evolutionary Rate Profiling (https://genome.ucsc.edu/cgi-bin/hgTrackUi?hgsid=2254050534_nBaaFJCLHSam3Ppo2AZXt1aqH2Dl&db=hg19&c=chr16&g=allHg19RS_BW)

GnomAD / ExAC: The Genome Aggregation Database / The Exome Aggregation Consortium (https://gnomad.broadinstitute.org/)

14KJPN / 8.3KJPN: Japanese Multi-Omics Reference Panel (https://jmorp.megabank.tohoku.ac.jp/)

ALFA: Allele Frequency Aggregator (https://www.ncbi.nlm.nih.gov/bioproject/PRJNA507278)

1000G_30x: 1000 Genomes Project phase 3: 30X coverage whole genome sequencing (https://www.internationalgenome.org/data-portal/data-collection)

KOREAN / KOVA: KOREAN population from KRGDB / Korean Variant Archive (<https://www.kobic.re.kr/kova/>)

dbSNP: The Single Nucleotide Polymorphism database (https://www.ncbi.nlm.nih.gov/snp/)

ClinVar: Public archive of relationships among sequence variation and human phenotype (<https://www.ncbi.nlm.nih.gov/clinvar/>)

| **Supplementary table 2.** The information of 7 mismatched mutations | | | | | | | | |  |  |  |  |
| --- | --- | --- | --- | --- | --- | --- | --- | --- | --- | --- | --- | --- |
| *OTOA* | | | | | | | | | | Paired region at *OTOAP1* | | |
| CHROM | POS | REF | ALT | CONSEQUENCE | IMPACT | SYMBOL | HGVSc | HGVSp | Exon | CHROM | POS | REF |
| chr16 | 21742179 | C | T | synonymous_variant | LOW | *OTOA* | NM_144672.4:c.2229C>T | NP_653273.3:p.Ala743Ala | 21/29 | chr16 | 22585305 | T |
| chr16 | 21747633 | A | C | missense_variant | MODERATE | *OTOA* | NM_144672.4:c.2353A>C | NP_653273.3:p.Thr785Pro | 22/29 | chr16 | 22563756 | C |
| chr16 | 21747639 | G | T | stop_gained | HIGH | *OTOA* | NM_144672.4:c.2359G>T | NP_653273.3:p.Glu787Ter | 22/29 | chr16 | 22563762 | T |
| chr16 | 21747697 | A | C | missense_variant | MODERATE | *OTOA* | NM_144672.4:c.2417A>C | NP_653273.3:p.Tyr806Ser | 22/29 | chr16 | 22563820 | C |
| chr16 | 21771873 | G | A | 3_prime_UTR_variant | MODIFIER | *OTOA* | NM_144672.4:c.*12G>A | . | 29/29 | chr16 | 22588015 | A |
| chr16 | 21771908 | A | G | 3_prime_UTR_variant | MODIFIER | *OTOA* | NM_144672.4:c.*47A>G | . | 29/29 | chr16 | 22588050 | G |
| chr16 | 21771945 | C | G | 3_prime_UTR_variant | MODIFIER | *OTOA* | NM_144672.4:c.*84C>G | . | 29/29 | chr16 | 22588087 | G |
